# Impact of sub-optimal HIV viral control on activated T-cells: An Earnest Sub study

**DOI:** 10.1097/QAD.0000000000003488

**Published:** 2023-01-20

**Authors:** Francesca I F Arrigoni, Moira Spyer, Patricia Hunter, Dagmar Alber, Cissy Kityo, James Hakim, Allen Matubu, Patrick Olal, Nicholas I Paton, A. Sarah Walker, Nigel Klein, the EARNEST trial team, E Agweng, P Awio, G Bakeinyaga, C Isabirye, U Kabuga, S Kasuswa, M Katuramu, C Kityo, F Kiweewa, H Kyomugisha, E Lutalo, P Mugyenyi, D Mulima, H Musana, G Musitwa, V Musiime, M Ndigendawan, H Namata, J Nkalubo, P Ocitti Labejja, P Okello, P Olal, G Pimundu, P Segonga, F Ssali, Z Tamale, D Tumukunde, W Namala, R Byaruhanga, J Kayiwa, J Tukamushaba, S Abunyang, D Eram, O Denis, R Lwalanda, L Mugarura, J Namusanje, I Nankya, E Ndashimye, E Nabulime, D Mulima, O Senfuma, G Bihabwa, E Buluma, P Easterbrook, A Elbireer, A Kambugu, D Kamya, M Katwere, R Kiggundu, C Komujuni, E Laker, E Lubwama, I Mambule, J Matovu, A Nakajubi, J Nakku, R Nalumenya, L Namuyimbwa, F Semitala, B Wandera, J Wanyama, H Mugerwa, A Lugemwa, E Ninsiima, T Ssenkindu, S Mwebe, L Atwine, H William, C Katemba, S Abunyang, M Acaku, P Ssebutinde, H Kitizo, J Kukundakwe, M Naluguza, K Ssegawa, Namayanja, F Nsibuka, P Tuhirirwe, M Fortunate, J Acen, J Achidri, A Amone, M. Chamai, J Ditai, M Kemigisa, M Kiconco, C Matama, D Mbanza, F Nambaziira, M Owor Odoi, A Rweyora, G. Tumwebaze, H Kalanzi, J Katabaazi, A Kiyingi, M Mbidde, M. Mugenyi, R Mwebaze, P Okong, I Senoga, M Abwola, D Baliruno, J Bwomezi, A Kasede, M Mudoola, R Namisi, F Ssennono, S Tuhirwe, G Abongomera, G Amone, J Abach, I Aciro, B Arach, P Kidega, J Omongin, E Ocung, W Odong, A Philliam, H Alima, B Ahimbisibwe, E Atuhaire, F Atukunda, G Bekusike, A Bulegyeya, D. Kahatano, S Kamukama, J Kyoshabire, A Nassali, A Mbonye, T M Naturinda, Ndukukire, A Nshabohurira, H. Ntawiha, A Rogers, M Tibyasa, S. Kiirya, D. Atwongyeire, A. Nankya, C. Draleku, D. Nakiboneka, D. Odoch, L. Lakidi, R. Ruganda, R. Abiriga, M. Mulindwa, F. Balmoi, S. Kafuma, E. Moriku, J Hakim, A Reid, E Chidziva, G Musoro, C Warambwa, G Tinago, S Mutsai, M Phiri, S Mudzingwa, T Bafana, V Masore, C Moyo, R Nhema, S Chitongo, Robert Heyderman, Lucky Kabanga, Symon Kaunda, Aubrey Kudzala, Linly Lifa, Jane Mallewa, Mike Moore, Chrissie Mtali, George Musowa, Grace Mwimaniwa, Rosemary Sikwese, Joep van Oosterhout, Milton Ziwoya, H Chimbaka, B Chitete, S Kamanga, T Kayinga E Makwakwa, R Mbiya, M Mlenga, T Mphande, C Mtika, G Mushani, O Ndhlovu, M Ngonga, I Nkhana, R Nyirenda, P Cheruiyot, C Kwobah, W Lokitala Ekiru, M Mokaya, A Mudogo, A Nzioka, A Siika, M Tanui, S Wachira, K Wools-Kaloustian, P Alipalli, E Chikatula, J Kipaila, I Kunda, S Lakhi, J Malama, W Mufwambi, L Mulenga, P Mwaba, E Mwamba, A Mweemba, M Namfukwe, E Kerukadho, B Ngwatu, J Birungi, MRC Clinical Trials Unit, N Paton, J Boles, A Burke, L Castle, S Ghuman, L Kendall, A Hoppe, S Tebbs, M Thomason, J Thompson, S Walker, J Whittle, H Wilkes, N Young, Monitors, C Kapuya, F Kyomuhendo, D Kyakundi, N Mkandawire, S Mulambo, S Senyonjo, Clinical Expert Review Committee, B Angus, A Arenas-Pinto, A Palfreeman, F Post, D Ishola, European Collaborators, J Arribas, R Colebunders, M Floridia, M Giuliano, P Mallon, P Walsh, M De Rosa, E Rinaldi, Trial Steering Committee, I Weller, C Gilks, J Hakim, A Kangewende, S Lakhi, E Luyirika, F Miiro, P Mwamba, P Mugyenyi, S Ojoo, N Paton, S Phiri, J van Oosterhout, A Siika, S Walker, A Wapakabulo, Data Monitoring Committee, T Peto, N French, J Matenga, Pharmaceutical companies, G Cloherty, J van Wyk, M Norton, S Lehrman, P Lamba, K Malik, J Rooney, W Snowden, J Villacian

**Affiliations:** https://ror.org/05gm41t98 JCRC Kampala, Uganda, (African trial co-ordinating centre); https://ror.org/02caa0269 IDI, Kampala, Uganda; JCRC, Mbarara, Uganda; JCRC Fort Portal, Uganda; San Raphael of St Francis Hospital, Nsambya, Uganda; JCRC Mbale, Uganda; JCRC Gulu, Uganda; JCRC Kabale, Uganda; JCRC Kakira, Uganda; https://ror.org/04ze6rb18 University of Zimbabwe Clinical Research Centre, Harare, Zimbabwe; Department of Medicine, University of Malawi College of Medicine; https://ror.org/03tebt685 Malawi-Liverpool-Wellcome Trust Clinical Research Programme, University of Malawi College of Medicine, Malawi; Mzuzu Central Hospital, Mzuzu, Malawi; Moi Teaching and Referral Hospital, Kenya; https://ror.org/03zn9xk79 University Teaching Hospital, Zambia; https://ror.org/05kvxgx64 The Aids Support Organisation (TASO), Uganda; Hospital La Paz, Madrid, Spain; https://ror.org/03xq4x896 Institute of Tropical Medicine, Antwerp, Belgium; ISS, Italy; ISS, Italy; https://ror.org/05m7pjf47 University College Dublin, Ireland; https://ror.org/05m7pjf47 University College Dublin, Ireland; https://ror.org/02f013h18 CINECA, Italy; https://ror.org/02f013h18 CINECA, Italy; a https://ror.org/02jx3x895 UCL, Great Ormond Street, Institute of Child Health London, UK; b Department of Pharmacy, https://ror.org/05bbqza97 Kingston University, London, UK; c https://ror.org/001mm6w73 MRC Clinical Trials Unit at University College London, London, UK; d https://ror.org/05gm41t98 Joint Clinical Research Centre (JCRC), Kampala, Uganda; e https://ror.org/04ze6rb18 University of Zimbabwe Clinical Research Centre, Harare, Zimbabwe; f Yong Loo Lin School of Medicine, https://ror.org/01tgyzw49 National University of Singapore, Singapore

**Keywords:** HIV-1, antiretroviral therapy, lymphocyte activation, viral load, immune reconstitution, immunophenotyping

## Abstract

**Objective:**

HIV viral load (VL) monitoring is generally conducted 6-12 monthly in low- and middle-income countries, risking relatively prolonged periods of poor viral control. We explored the effects of different levels of loss of viral control on immune reconstitution and activation.

**Design:**

208 participants starting Protease Inhibitor (PI)-based second-line therapy in the EARNEST trial (ISRCTN37737787) in Uganda and Zimbabwe were enrolled and CD38^+^/HLA-DR^+^ immunophenotyping performed (CD8-FITC/CD38-PE/CD3-PerCP/HLA-DR-APC; centrally gated) in real-time at 0, 12, 48, 96 and 144 weeks from randomisation.

**Methods:**

Viral Load (Viral load (VL) was assayed retrospectively on samples collected every 12-16 weeks and classified as (1) continuous suppression (<40 copies/ml throughout); (2) suppression with transient blips; (3) low-level rebound (two or more consecutive VL >40, <5000 copies/ml); (4) high-level rebound/non-response (two or more consecutive VL >5000 copies/ml).

**Results:**

Immunophenotype reconstitution varied between that defined by numbers of cells and that defined by cell percentages. Furthermore, VL dynamics were associated with substantial differences in expression of CD4^+^ and CD8^+^ cell activation markers, with only individuals with high-level rebound/non-response (>5000 copies/ml) experiencing significantly greater activation and impaired reconstitution. There was little difference between participants who suppressed consistently and who exhibited transient blips or even low-level rebound by 144 weeks (p>0.2 vs suppressed consistently).

**Conclusion:**

Detectable viral load below the threshold at which WHO guidelines recommend that treatment can be maintained without switching (1000 copies/ml) appear to have at most, small effects on reconstitution and activation, for patients taking a PI-based second-line regimen.

## Introduction

In patients failing first line antiretroviral therapy (ART), second line therapy has proven successful in decreasing HIV viral load. In one such study, the impact of three different Protease Inhibitor (PI)-based second-line regimens on adults/adolescents was studied in the Europe-Africa Research Network for Evaluation of Second Line Therapy (EARNEST) trial[1]. Despite significantly lower viral load suppression with the monotherapy strategy to 96 weeks of therapy, similar increases in CD4+ counts were observed across the three randomised arms with little to differentiate them at 96 weeks, or 144 weeks when a substantial proportion of the PI mono group had moved to PI/NRTI [2].

It remains unclear why differences in the effectiveness of ART controlling viral loads did not appear to regulate circulating CD4 counts. It is possible that any influence on total CD4 cell number is hidden by an excess of cells generated at other sites, including lymphoid tissues and transferred at higher rates to the circulation. It is also possible that the homeostatic processes which regulate circulating CD4 cells are modified differentially between ART regimes, ultimately leading to similar levels of peripheral CD4 cells. The impact of this might influence HIV disease persistence, reservoirs and progression, even in the context of ART, and is critical for developing optimal or novel treatment strategies.

HLA-DR and CD38 can be used as markers of immune activation [3] and are found at high levels with progression of HIV disease [4]. A study has suggested that differing levels of viral control may be key in influencing T cell activity [5]. However, exactly how this may be operating in the EARNEST study is unclear. For example, while both HLA-DR and CD38 may indicate general levels of immune activation, expression of CD38 on CD4^+^ cells can also indicate higher levels of HIV DNA within CD4 reservoirs, and may contribute to HIV latency[6], that may be important at driving HIV progression.

In this sub-study we tested the hypothesis that within the EARNEST trial, the type of second line regimen adopted could influence the immunological responses, examining the impact that viral load has on immunophenotype, and we highlight the importance of incorporating this approach into future ART trials.

## Methods

### Study Design and Participants

#### Overall EARNEST trial design

In the Europe-Africa Research Network for Evaluation of Second Line Therapy (EARNEST) trial (ISRCTN37737787), 1277 adults/adolescents failing first-line antiretroviral therapy (ART) were randomised to three different second-line regimens: a ritonavir-boosted protease inhibitor (standardised to lopinavir/ritonavir) plus two or three new or recycled nucleoside reverse transcriptase inhibitors (PI/NRTI), a boosted protease inhibitor plus raltegravir (PI/ RAL), or a boosted protease inhibitor plus raltegravir for 12 weeks followed by PI monotherapy (PI mono). Following review of week 96 data by the Data Monitoring Committee, patients still in the trial on PI monotherapy moved to PI/NRTI combination therapy.

#### Substudy design

All patients recruited to the EARNEST trial at the Joint Clinical Research Centre in Kampala, Uganda and University of Zimbabwe Clinical Research Centre in Harare, Zimbabwe after 20 October 2010 and 3 February 2011, respectively were eligible for this immunology substudy. There were no specific exclusion criteria. The substudy recruited 126/131 (96%) from JCRC and 82/94 patients (87%) from UZCRC joining the main trial after these respective dates. The substudy was included in the main trial protocol which was approved by ethics committees in all participating countries and the UK. All participants (and caregivers of adolescents <18 years) provided written informed consent.

CD4 and CD8 were assayed using standard flow cytometry as part of the main trial. Within the substudy, activation markers were assayed on the same samples taken at screening (1-7 days before enrolment and randomisation when participants switched to second-line therapy), then at week 12, week 48, week 96 and week 144 on second-line therapy. Due to lack of available reagents, most week 96 samples could not be tested in Zimbabwe (Supplementary Figure 1). Samples were refrigerated and immunostaining was carried out within 24 hours of collection. Stained and fixed samples were analysed within 4 days. Briefly, a cocktail of fluorescently tagged antibodies, CD8-FITC/CD38-PE/CD3-PerCP/HLA-DR-APC (Becton Dickenson), was added to 100µl whole blood and data acquired on a BD FACSCalibur flow cytometer. The 4 markers chosen, CD3, CD8, CD38 and HLA DR, were gated within a specific window of size and granularity, and as phenotypes that when expressed together cannot be found on red blood cells or on other cells aside from T cells which allow consideration as markers activation.

The analysis of the flow cytometry data for the entire cohort across all the sites following its acquisition, was carried out by 1 person, limiting discrepancies in measurement variability.

Cell populations were gated by SSC, FSC then CD3, of which cells were then gated according to expression of CD8^+^ or CD8^-^. The CD8^-^ were assumed to be CD4^+^ cells. Within the CD8^+^ and assumptive CD4^+^ populations, further subdivision into CD38^+^/HLA-DR^-^, CD38^+^/HLA-DR^+^, CD38^-^/HLA-DR^-^, and CD38^-^/HLA-DR^+^ quadrants highlighted the proportions of activated cells.

Analysis was undertaken centrally using Cellquest Software (Becton Dickenson). HIV viral load (VL) was assayed on stored specimens retrospectively in batches at enrolment and weeks 4, 12, 24, 36, 48, 64, 80, 96, 112, 128 and 144 (Abbott RealTime HIV-1 (m2000sp) assay, Abbott Molecular Inc., USA. VL response to each timepoint where activation markers were measured was characterised as suppressed consistently if participants had a VL <40 copies/ml by week 12 or a >1 log10 drop in VL and were <40 copies/ml at all subsequent measurements; or as suppressed with transient blips if only isolated VL measurements were ≥40 copies/ml (as described in ^[5]^). Those not re-suppressing <40 copies/ml but remaining <5000 copies/ml were classified as low-level rebound, whereas those whose VL measurements were consistently ≥5000 copies/ml or who never suppressed were classified as high-level rebound or non-response. The choice of 5000 copies/ml was designed to identify rebound above levels at which current WHO guidelines recommend switching, and divide unsuppressed VL in the trial into approximate 0-2 and 2-4 log_10_ increases above the limit of quantification (40-5000 and 5000-500000 copies/ml respectively, reflecting previous definitions [5, 7].

#### Analysis

The mean and 95% confidence intervals for percentages of T-cells from the quadrant populations, including total CD38^+^ and total HLA-DR^+^, were calculated; overall, by randomised group and VL response group, at each timepoint, and compared between groups using ANCOVA at each time point (adjusted for baseline value). Pairwise comparisons between groups were interpreted only if the overall test across all groups reached p<0.05 (analogous to a closed testing procedure). As well as analysing cell percentages, we also quantified absolute cell numbers by normalising to the total and quadrant populations of CD4^+^ and CD8^+^ cell numbers from the main flow cytometry assays.

## Results

The 208 adults included in this immunology substudy were randomly assigned to PI/NRTI (n=69), PI/RAL (n=71) and PI mono (n=68); (median age 39 [IQR 34-46] years, median VL 50660 [IQR 18789-183568] copies/ml, mean CD4 112 cells/mm^3^ at baseline (switch to second-line)). Overall, over half of the participants had available immunophenotyping over the course of the study (208, 140 (67%), 183 (88%), 106 (51%) and 147 (71%) at baseline, 12, 48, 96 and 144 weeks, respectively), with most missing data due to lack of reagents in Zimbabwe for the week 96 visit (Supplementary Figure 1).

CD4^+^ reconstitution was similar to that in the trial overall [8], with CD4^+^ % increasing steadily from baseline to plateau at 96 weeks (mean 20% (95%CI 18-21) compared with mean 21% (95%CI 20-22) at 144 weeks) (Table 1). Absolute CD4^+^ cell numbers rose throughout the 144 weeks (to mean 408 cells/mm^3^ at 144 weeks (95%CI 378-438)) (Table 1). Similarly to the trial overall, the percentage of CD8^+^T-cells decreased on second-line therapy (144 vs 0 weeks; p<0.0005) while CD8^+^ numbers significantly increased (144 vs 0 weeks; p<0.0005).

**Figure 1:**
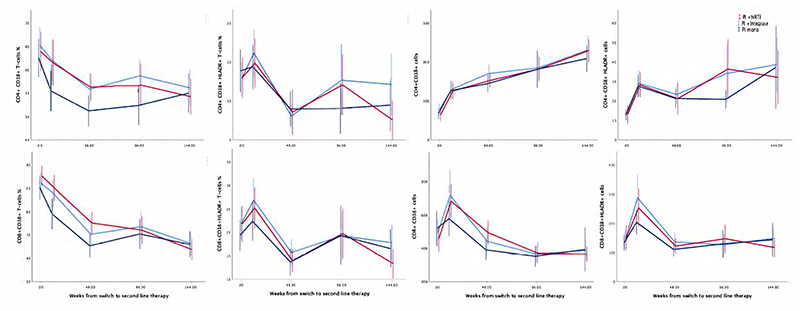
Number and proportion of activated CD4 and CD8 cells over time on second-line ART Note: Showing mean plus 95% confidence interval for each randomised group at each timepoint.

In terms of the specific activation markers, there was no evidence that either the numbers or proportions of activated T-cells varied between PI/RAL vs PI/NRTI at week 144 (p>0.06), or across all three randomised groups over weeks 12-144 (p>0.3 adjusting for baseline values), with the single exception of CD4^+^CD38^+^ T-cell % which was lower in PI monotherapy at 12 weeks (P=0.04) (Figure 1, Supplementary Table 1). However, as all PI monotherapy received raltegravir induction therapy through to week 12 this was a chance finding. Subsequent analyses therefore pooled randomised groups.

Overall, the percentages of activated CD4^+^ T-cells (CD4^+^CD38^+^ and CD4^+^CD38^+^HLA-DR^+^) decreased on second-line therapy to reach a steady state by 48 weeks (48 vs 0 weeks; p<0.0005), with no evidence of subsequent changes from week 48 to 144 (p>0.39) (Figure 1). However, when normalised to the circulating numbers of CD4^+^ T-cells, numbers of activated CD4^+^CD38^+^ cells actually increased over 144 weeks (144 vs 0 weeks; p<0.0005) as did CD4^+^CD38^+^HLA-DR^+^ (p<0.0005) (Figure 1, Table 1). Activated CD8^+^ T-cells (both number and percentage CD8^+^CD38^+^ and CD8^+^CD38^+^HLA-DR^+^) generally decreased continuously over follow-up or decreased to week 48 then plateaued (Figure 1, Table 1), however in numbers of circulating CD8^+^CD38^+^ and CD8^+^CD38^+^HLA-DR^+^ cells/mm^3^ this occurred following an initial increase from baseline to week 12. Similar but smaller effects for CD8^+^CD38^+^HLA-DR^+^ percentages were observed, potentially due to decreased rates of cell death on ART.

### VL suppression

**Figure 2:**
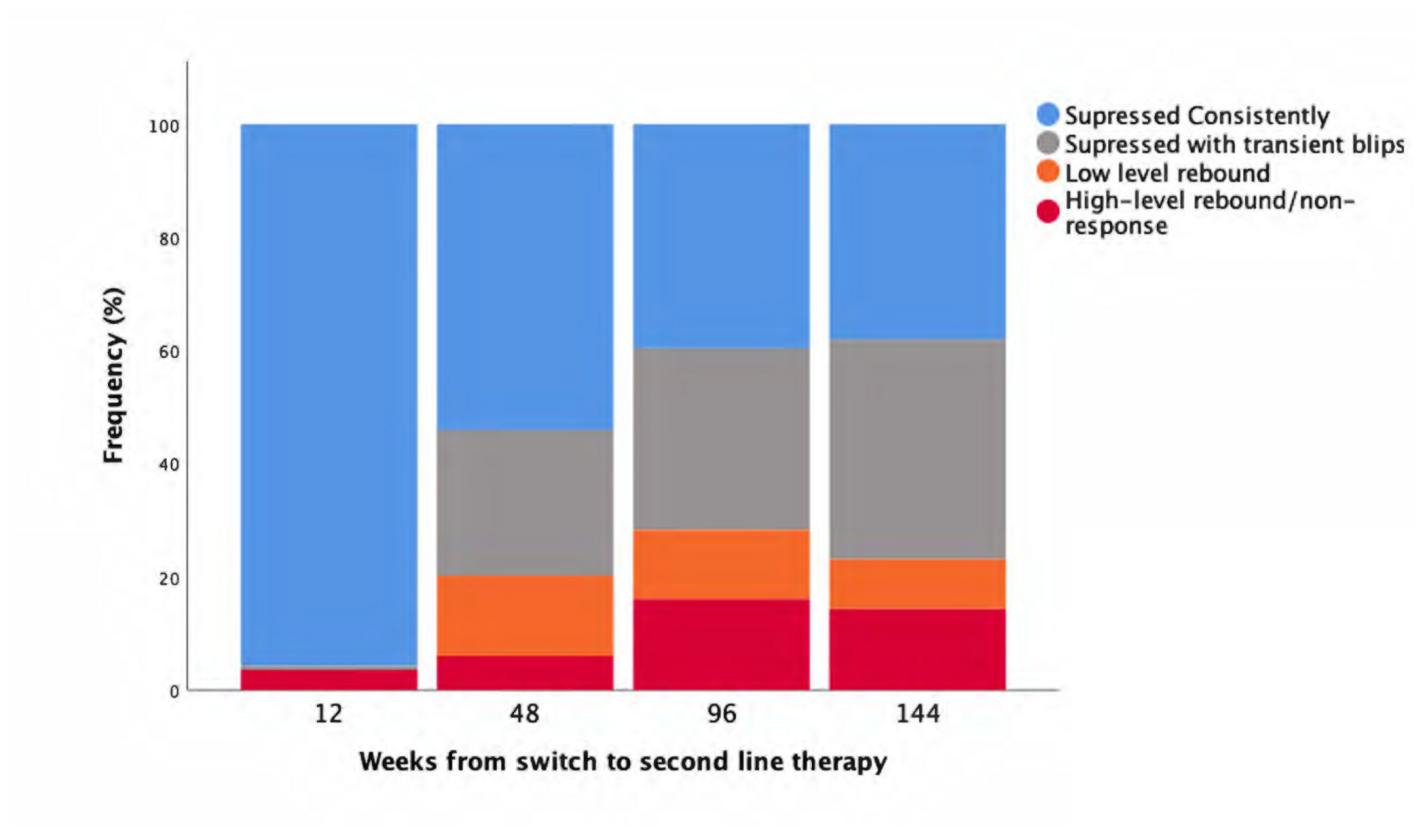
Viral load responses over time on second line therapy

To investigate the influences of VL control on T-cell activation, we classified individuals according to their complete VL trajectories (including VLs assayed at intermediate timepoints to when immunophenotyping was performed) [5] (Figure 2, Supplementary Table 2). After 12 weeks of second-line therapy, 134 (96%) of the 140 substudy participants with immunophenotyping attained an initial VL response of <40 copies/ml. However, by week 144 only 56 (38%) of the 147 participants with immunophenotyping had suppressed consistently throughout 144 weeks, with 57 (39%) suppressed with transient blips, 13 (13%) with low-level rebound and 21 (14%) with high-level rebound or never suppressing VL. At week 144, median [IQR] weeks spent in each of these categories was 140 (140-140), 96 (48-120), 120 (64-120) and 80 (48-96), respectively.

### Dynamic changes in CD4+ and CD4+ cell populations by VL response

**Figure 3:**
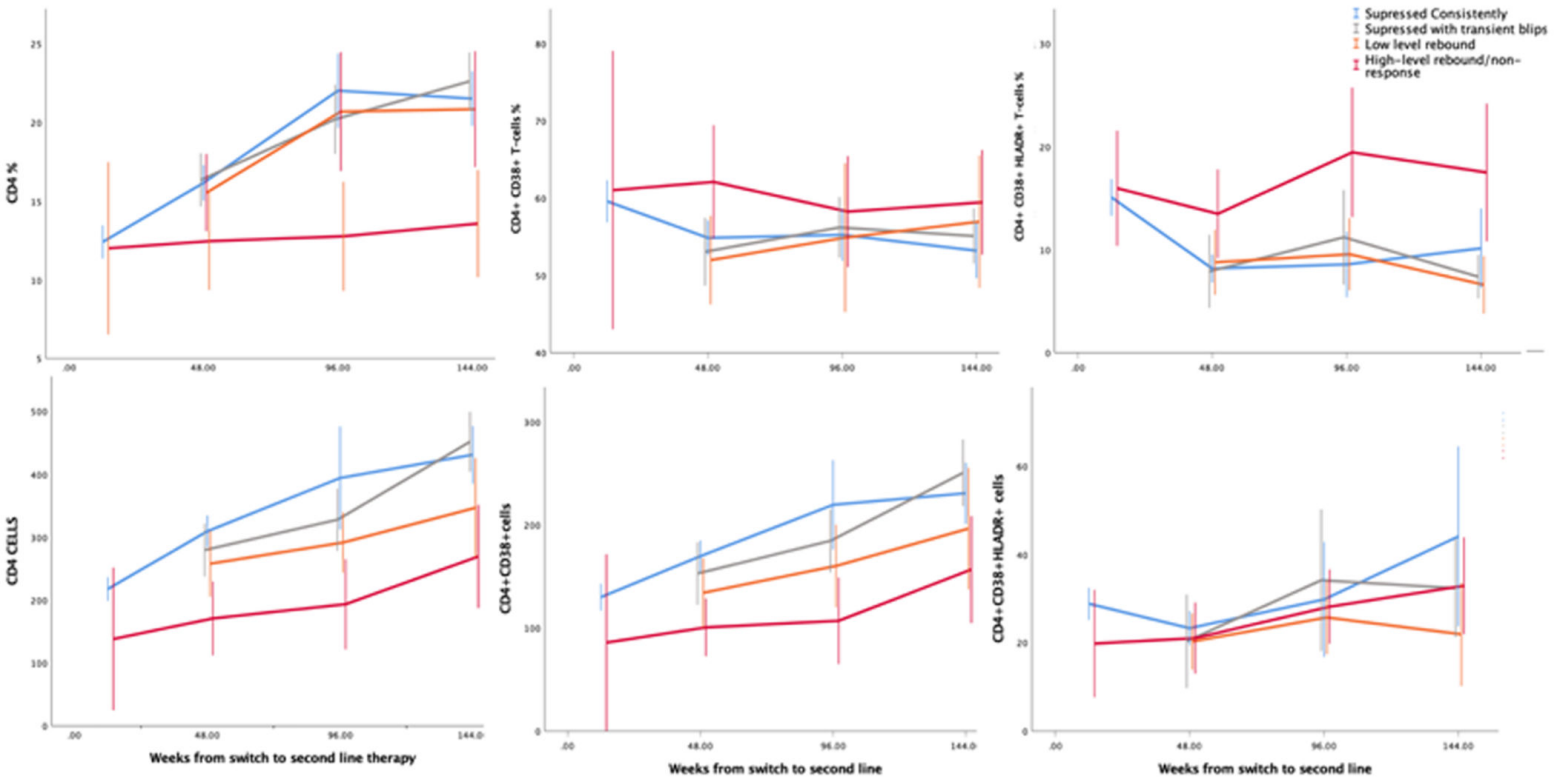
Changes in CD4 and activated CD4 sub-populations over time, expressed both as percentages and normalised to circulating CD4 levels. Note: showing mean plus 95% confidence interval for each group at each timepoint.

Only participants with high-level rebound failed to increase their percentage of CD4^+^T-cells and decrease their percentage of CD4^+^CD38^+^HLA-DR^+^, over 144 weeks (Figure 3, Supplementary Table 3), with significantly lower CD4+T-cell percentage at week 144 than those suppressed consistently (mean 14% (95%CI 10-17) vs 22% (95% CI 20-23), respectively p<0.0005). In contrast, CD4+T-cell percentages were similar in those suppressed consistently and in those suppressed with transient blips or with low-level rebound, over time and at week 144 (mean 23% (95%CI 21-25) and 21% (95% CI 17-25) respectively). Absolute CD4^+^ cell numbers however, showed clear gradations with level of virus, despite steadily increasing from initiation through week 144, even in those with high-level rebound (p=0.01). Changes in the CD4^+^:CD8^+^ ratio in the diverse VL responses mirrored those for CD4^+^%, with no evidence of change over time in those with high-level rebound/non-response, compared to increases in other groups (Supplementary Figure 2, Supplementary Table 3).

**Figure 4:**
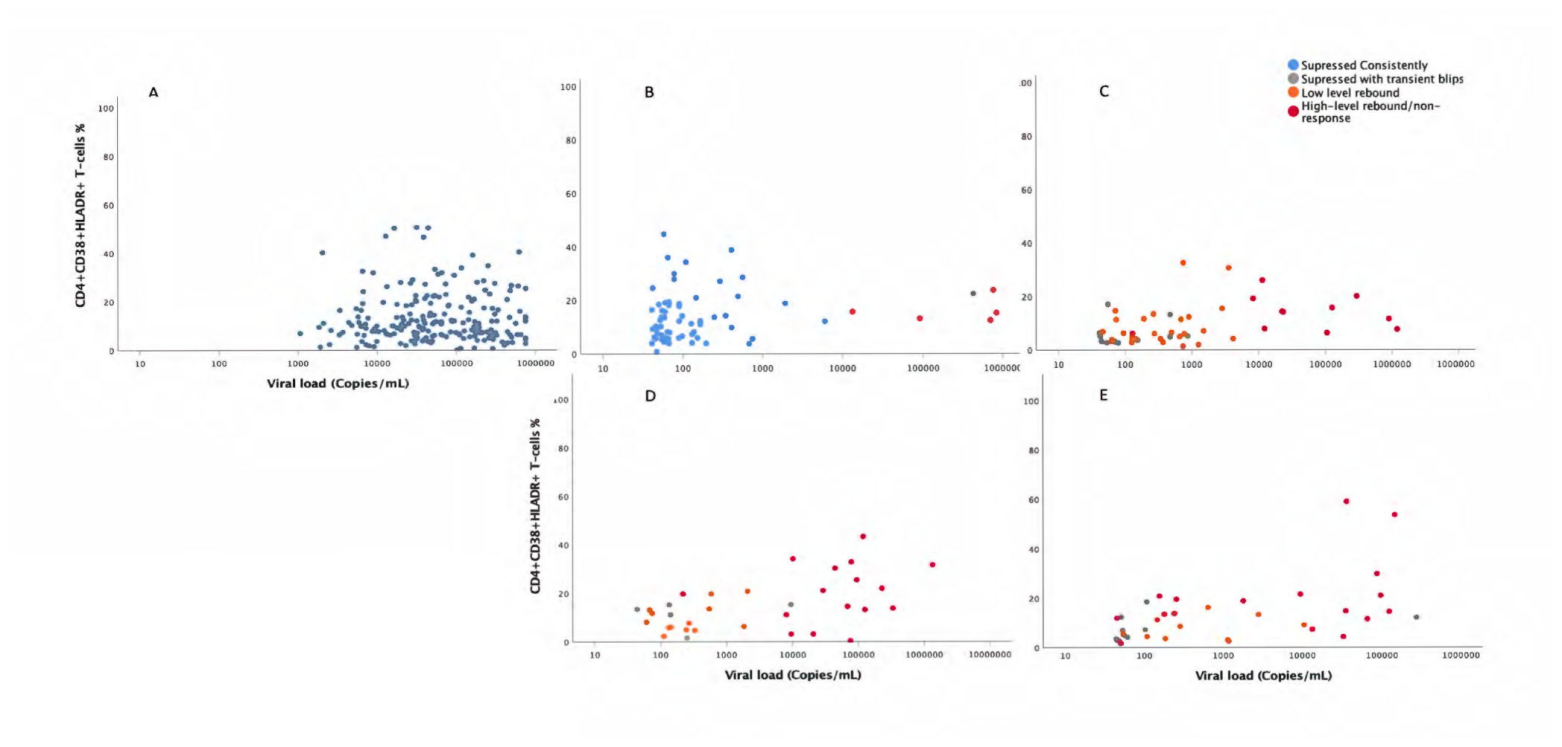
CD4/CD38/HLADR expression and viral load, by VL response at baseline (A), 12 weeks (B), 48 weeks (C), 96 weeks (D) and 144 weeks (E) for viral loads >40 copies/ml. Note: Definition of suppressed consistently at 12 weeks includes VL response

There was no evidence that VL group affected the percentage of CD4^+^CD38^+^ T-cells or the numbers of CD4^+^CD38^+^HLA-DR^+^ cells (p>0.2 comparing the four categories at all timepoints). However, participants with high-level rebound/non-response had consistently lower numbers of CD4^+^CD38^+^ cells and higher percentages of CD4^+^CD38^+^HLA-DR^+^ T-cells compared with cells from consistently suppressed individuals (Figure 4 Supplementary Table 3). In contrast, there was no evidence of differences between those suppressed consistently, suppressed transiently or with low-level rebound in the percentages of CD4^+^CD38^+^HLA-DR^+^ (or CD4^+^CD38^+^) cells, and the absolute numbers of CD4^+^CD38^+^ cells showed a similar gradation with level of virus to CD4+T-cells.

### Dynamic changes in CD8+ and CD8+ cell populations by VL response

**Figure 5:**
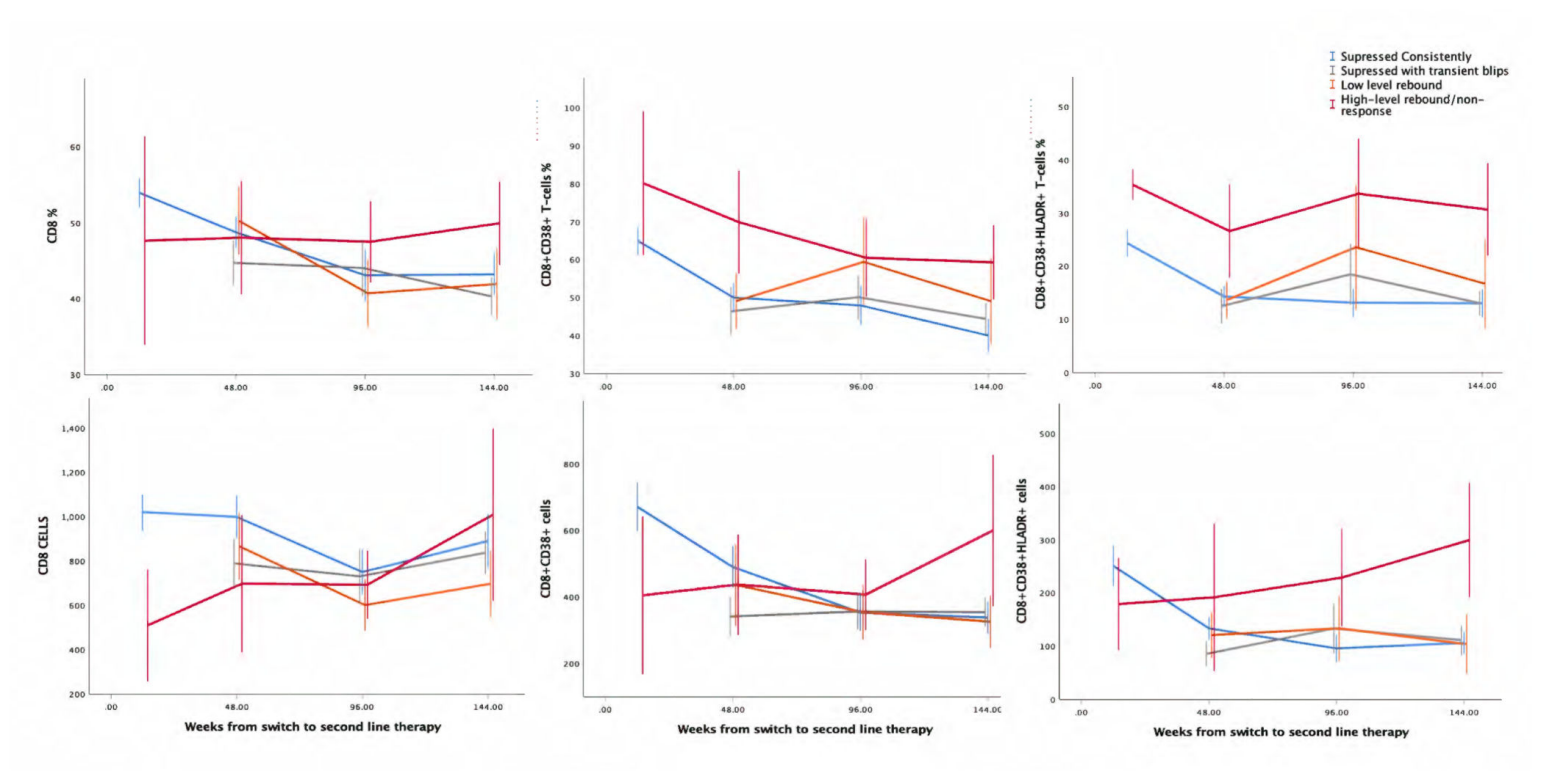
Changes in CD8 and activated CD8 sub-populations over time, expressed both as percentages and normalised to circulating CD8 levels. Note: Showing mean plus 95% confidence interval for each group at each timepoint.

Again, only participants with high-level rebound/non-response failed to decrease their percentage of CD8^+^ T-cells (Figure 5, Supplementary Table 3), with CD8^+^ T-cell percentage decreasing in all other groups. CD8^+^ cell numbers tended to increase in participants with high-level rebound/non-response, whereas there was no evidence of any difference in cell number over follow-up for the other three groups. Both the percentage and number of CD8^+^CD38^+^ and CD8^+^CD38^+^HLA-DR^+^ cells were higher in participants with high-level rebound/non-response compared to those who were suppressed consistently (Figure 5, Supplementary Table 3). However, all activation cell numbers and percentages were more similar in those suppressed consistently, suppressed with transient blips and with low-level rebound.

### Relationship between expression of CD38^+^ and HLA-DR^+^ in CD8^+^ cells

The expression of CD8^+^CD38^+^HLA-DR^+^ as a % of T-cells in all patients was most strongly correlated with plasma viral load levels 144 weeks after starting second-line therapy (Spearman’s rho=0.52, p=0.001). In addition a significantly greater expression of CD8^+^CD38^+^HLA-DR^+^ was found in individuals with high-level rebound/non-response than in those suppressed consistently (p<0.0005) (Supplementary Table 3).

## Discussion

In this substudy, despite the inferior virological response to PI monotherapy, all second-line regimens were associated with good immunological responses overall [1]. Circulating numbers of CD4^+^ cells continued to normalise regardless of regimen and had still not reached a steady state by week 144.

However, we found a strong influence of viraemia on both immune reconstitution in general, and markers of immune activation in particular. However, of note, only those with high-level (VL) rebound/non-response had markedly different immune trajectories. Whilst the specific trajectories varied according to whether T cell percentages or numbers were considered, as previously reported [9], the inference was consistent, namely that there were many fewer, if any, differences between those suppressed consistently, suppressed with transient blips or with low-level rebound; whereas those with high-level rebound/non-response (>5000 copies/ml) were significantly impaired in immune reconstitution and activation.

Increases in CD4^+^ alongside parallel decreases in CD8^+^ occurred in all groups other than those with high-level rebound/non-response, emphasising the homeostatic balance in T cell production that has been strongly associated with HIV outcomes [10] including viraemia [11] and redressed following therapy. This was lower than found in other groups on therapy [10, 12], plausibly consistent with the relatively high degree of immune-suppression when failing first-line and starting second-line therapy.

Whilst important to consider that other Tcells such as T regs, exhausted T cells and CD4^-^ exist within the CD3^+^/CD8^-^ population, given that the number of these cells were not of significance to alter the patterns found in our data or change the conclusions drawn they remained labelled as CD4 ^+^.

Double expression of CD38^+^HLA-DR^+^ on CD4^+^ cells was examined. Associated with increased T cell activity during active HIV infection [3] and predictors of disease progression in untreated patients [4], significantly elevated percentages of CD38^+^HLA-DR^+^ were expressed in CD4^+^ cells in those individuals with high-level rebound/non-responses. HLA-DR^+^ expression is indicative of T cell activation [13] whilst CD38 has numerous cellular functions involved with cell metabolism[14] and activity, and is expressed in up to 30% of CD4^+^ cells in healthy adults [13]. Elevated in HIV, CD4^+^CD38^+^ declines when patients demonstrate successful viral control following combination ART [15].

Co-expression of CD8^+^CD38^+^HLA-DR^+^ is reported to be indicative of both activation and proliferation of CD8^+^ T-cells, reflecting strong antiviral function by exhibiting higher effector functions including proliferation, cytotoxicity and cytokine production [16, 17] however their expression is not co-dependent ^[18]^. During hyperacute HIV infection, up to 77% of peripheral CD8+ cells express both CD38^+^ and HLA-DR^+^ [19], contributing towards the setpoint for an individual. However, during chronic viral infections maintaining these levels of CD38^+^HLA-DR^+^ leads to increased expression of molecules related to exhaustion and apoptosis and both elevated percentages and absolute number of CD38^+^HLA-DR^+^ predict the progression to AIDS [20].

In individuals with high-level rebound/non-response unlike the other groups, CD8^+^CD38^+^HLA-DR^+^ continued to be strongly associated with viral load over 144 weeks and when normalised to circulating CD8^+^ cells, CD8^+^CD38^+^HLA-DR^+^ increased in number over time, which might suggest that as viral load comes under control, HLA-DR^+^/CD38^+^ is no longer co-expressed [21]. The strong correlation of CD8^+^CD38^+^HLA-DR^+^ with viral load in participants with high-level non-response/rebound by 144 weeks from initiation of second-line therapy has been associated with a poorer outcome [22] although which is the driving factor is unclear.

In those with high-level rebound/non-response more than half of CD8^+^ cells remained CD38 positive at week 144 although the percentages of CD8^+^ cells expressing CD38^+^ consistently decreased over the duration of the trial independent of viral load control.

CD38^+^ and HLA-DR^+^ expression are considered markers of activation that, either exclusively or co-expressed, correlate with viral load in non-controllers [23], although qualitative differences between HIV-1 specific CD8^+^ T cell responses, such as cytokine release, can define responses of controllers and those with progressive disease [24]. Elevated CD8^+^CD38^+^ and CD8^+^HLA-DR^+^ expression persists throughout HIV infection and has prognostic significance for progression onto AIDS [23, 25].

Over the duration of the trial, whilst the percentages of CD4^+^CD38^+^ and CD4^+^ CD38^+^HLADR^+^ T-cells decreased in all groups except high-level rebound, numbers of circulating CD4^+^CD38^+^ increased in all VL response groups when normalised to an increasing population of circulating CD4^+^ T-cells. Thus, although the percentage of activated cells decreased with functioning ART, this was offset by an overall increase in T cell number leading to a larger pool of active cells. Therefore in absolute terms, it could be argued that ART does not lead to reduced activation. Interestingly, and potentially important, CD8^+^ cells responded more quickly to fluctuations in viral replication [15], and generally demonstrated larger differences than CD4^+^ parameters when comparing the high-level rebound/non-response group and other groups. This may be an important factor for long term survival of this population.

In summary, this study demonstrates that the main drivers of immune reconstitution and activation are most likely viral, rather than being driven by specific ART combinations. Immune reconstitution is impaired in high level rebound and non-responsive individuals with viral loads above 5000 copies/ml, which is well above the WHO guidelines recommend level for switching. In contrast, provided patients remain on ART, transient blips and low-level rebound have at most small effects on reconstitution and activation. These findings suggest that the current WHO viral load threshold for switching to second-line of 1000 copies/ml should avoid the most deleterious effects of high-level rebound, given that it is mostly identified through annual viral load monitoring in many low and middle income settings.

### Limitations

In labelling our cells we have classified those CD3^+^ CD8^-^ as CD4^+^ cells and acknowledge that this population of CD8^-^ may be CD4^-^/CD8^-^. We maintain that as a percentage of cells analysed over time, our labelled CD8^-^ follow the same independently analysed patterns of total CD4^+^ count reported previously [2] and have therefore maintained the labelling of CD8^-^ as CD4^+^. In the analysis of the subsets, the significantly elevated percentages of CD38^+^ HLADR^+^ in CD8^-^ cells in patients with high viral load in this study are likely to be CD8^-^CD4^+^ and not CD8^-^CD4^-^ as this population decreases with high viral load [26].

## Supplementary Material

Supplementary Figure 1

Supplementary Figure 2

Supplementary Table 1

Supplementary Table 2

Supplementary Table 3

## Figures and Tables

**Table 1:** Cell numbers and subpopulations over 144 weeks on second-line therapy.

	Weeks from switch to second-line therapy			p	Weeks from switch to second-line therapy		p	p	p
Weeks on Second Line Therapy	0	12	48	Week 48 V 0	96	144	week 144 v 0	week 144 v 12	week 144 v 48
**Numbers with results**	208	140	183		106	147		ANCOVA	adjusted for BL
**CD4 cells/mm^3^**	112 (98 to 125) N=193	213 (194 to 232)	286 (267 to 305) N=182	p<0.0005	328(288 to 367)	408 (378 to 438)	p<0.0005	p<0.0005	p<0.0005
**CD4%**	10 (9 to 11) N=193	12 (11 to 13)	16 (15 to 17) N=182	p<0.0005	20 (18 to 21)	21 (20 to 22)	p<0.0005	p<0.0005	p<0.0005
**CD4+ CD38+ T-cells %**	64 (62 to 66)	60 (57 to 62)	55 (53 to 56)	p<0.0005	56 (54 to 58)	55 (53 to 57)	p<0.0005	p=0.001	p=0.85
**CD4+CD38+HLADR+ T-cells %**	13 (12 to 15)	15 (13 to 17)	9 (7 to 10)	p<0.0005	11 (9 to 14)	10(8 to 12)	p=0.003	p<0.0005	p=0.39
**CD4+CD38+ cells**	69 (60 to 78) N=193	127 (114 to 140)	156 (144 to 168) N=182	p<0.0005	183 (161 to 205)	224 (205 to 243)	p<0.0005	p<0.0005	p<0.0005
**CD4+CD38+HLADR+ cells**	13 (11 to 15) N=193	28 (25 to 32)	22 (18 to 25) N=182	p<0.0005	30 (23 to 38)	36 (27 to 45)	p<0.0005	p=0.052	p<0.0005
**CD8 Cells**	690 (627 to 753) N=193	999 (917 to 1081)	908 (842 to 974) N=182	p<0.0005	716 (655 to 777)	867 (787 to 947)	p<0.0005	p<0.0005	p=0.361
**CD8 %**	58 (57 to 60) N=193	54 (52 to 56)	48 (46 to 49) N=182	p<0.0005	44 (42 to 46)	43 (41 to 45) N=146	p<0.0005	p<0.0005	p<0.0005
**CD8+CD38+ T-cells %**	73 (70 to 76)	66 (62 to 69)	50 (47 to 53)	p<0.0005	52 (49 to 55)	45 (42 to 48)	p<0.0005	p<0.0005	p=0.02
**CD8+CD38+HLADR+ T-cells %**	21 (19 to 23)	25 (22 to 27)	14 (13 to 16)	p<0.0005	19 (16 to 23)	16 (14 to 18)	p<0.0005	p<0.0005	p=0.66
**CD8+CD38+ cells**	488 (438 to 537) N=193	661 (590 to 732)	441 (399 to 483) N=182	p=0.094	362 (330 to 395)	378 (336 to 420)	p<0.0005	p<0.0005	p=0.04
**CD8+CD38+HLADR+ cells**	143 (122 to 163) N=193	249 (212 to 285)	123 (107 to 139) N=182	p=0.067	134(109 to 159)	134 (112 to 157)	p=0.104	p<0.0005	p=0.53
**CD4:CD8 ratio**	0.2 (0.2 to 0.2) N=193	0.3 (0.2 to 0.3)	0.4 (0.3 to 0.4) N=182	p<0.0005	0.5 (0.4 to 0.5)	0.5 (0.5 to 0.6)	p<0.0005	p<0.0005	p<0.0005

Note: showing mean and 95% confidence interval for each randomised group at each timepoint. 208 patients were immunophenoptyped at baseline; 193 patients had CD4 and CD8 data on the same sample as immunophenotyping at baseline (15 had CD4 and CD8 on screening sample only)
