# Supplementary figures and images for: Impact of sub-optimal HIV viral control on activated T-cells: An Earnest Sub study
Source: AIDS. Author manuscript; Available in PMC 2024 Dec 9. (PMC7617099; doi:10.1097/QAD.0000000000003488)

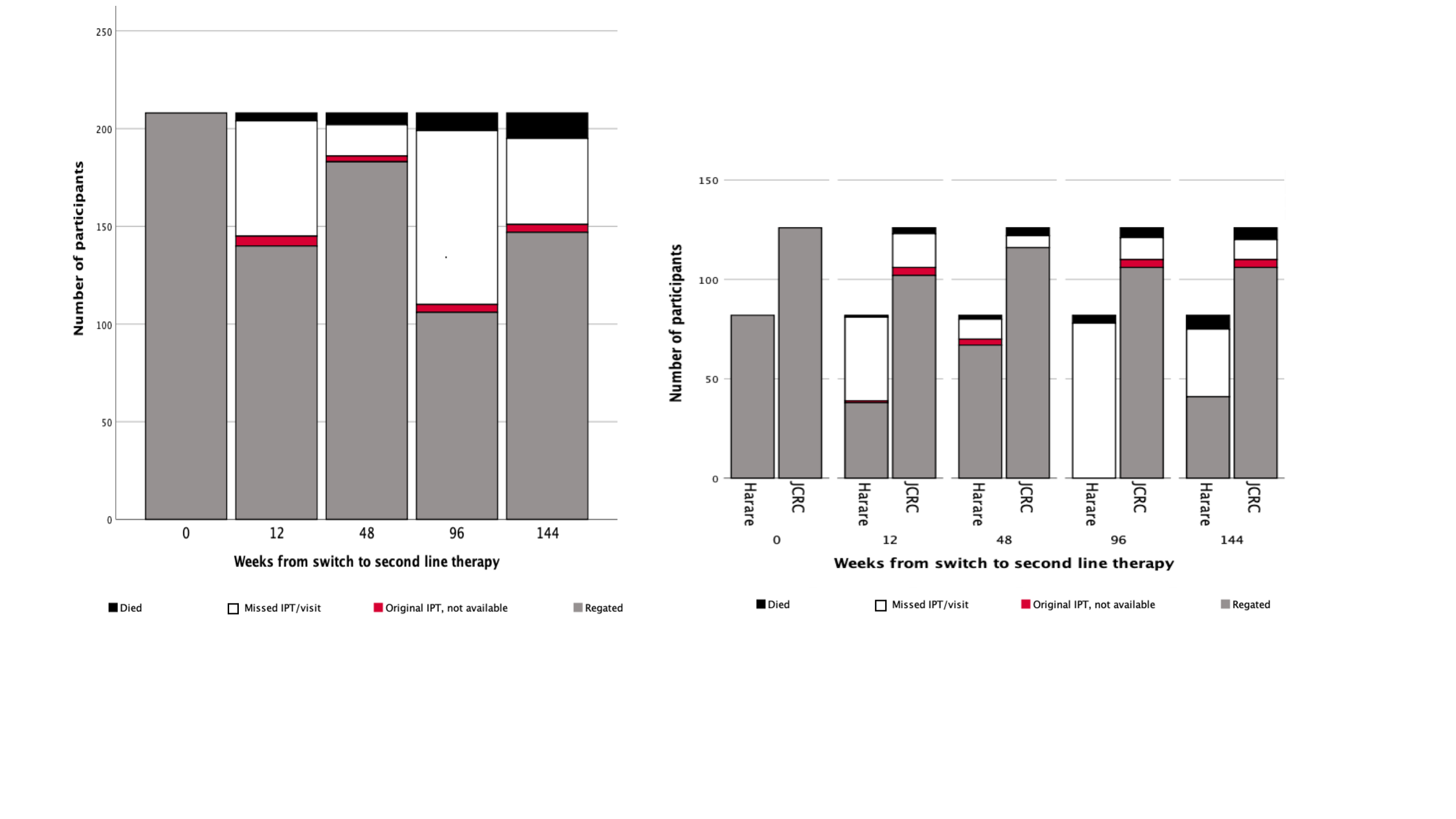

Supplement: Supplementary Figure 1 [file EMS200168-supplement-Supplementary_Figure_1.tiff]

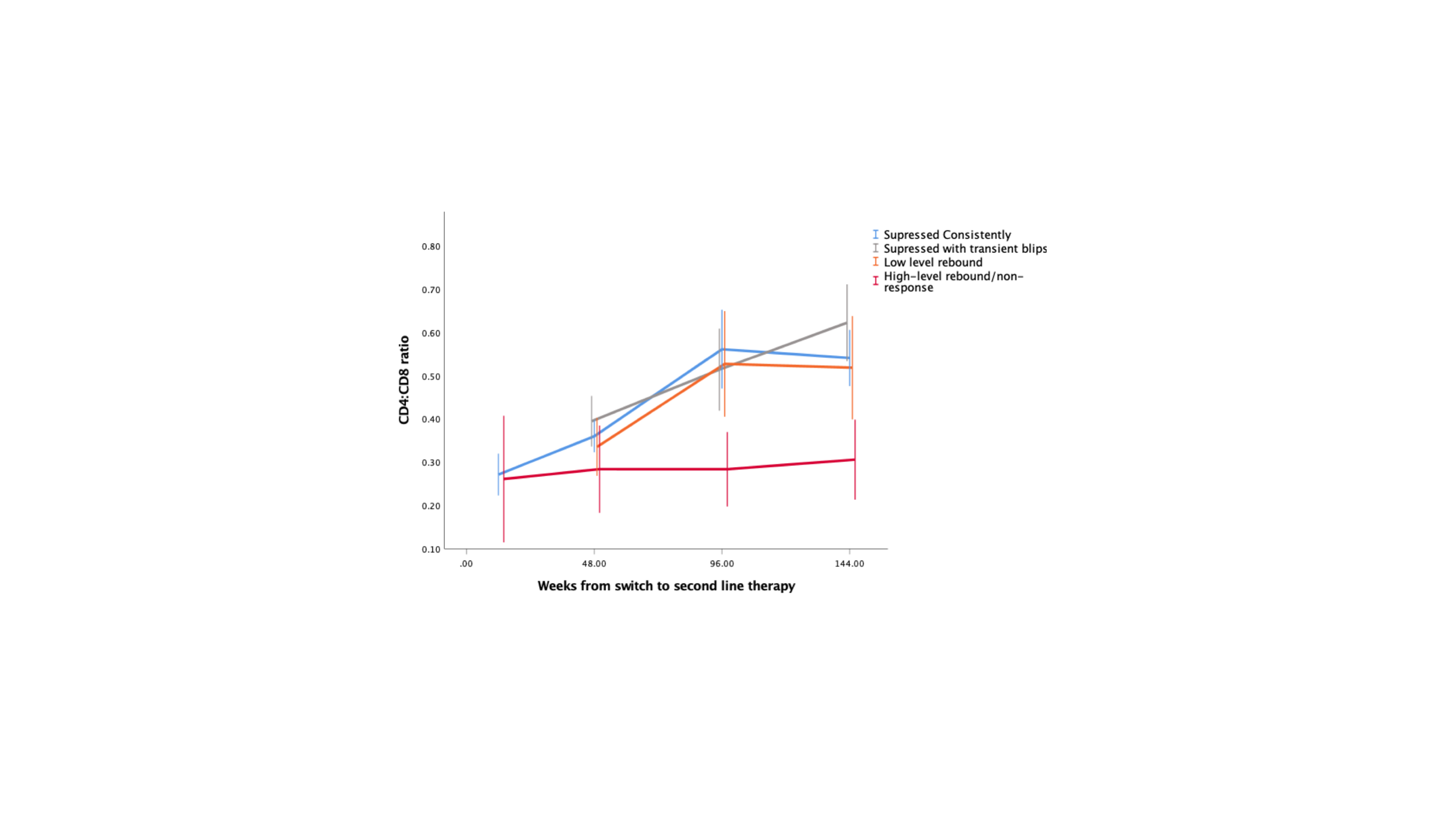

Supplement: Supplementary Figure 2 [file EMS200168-supplement-Supplementary_Figure_2.tiff]
