# Supplementary material for: Impact of sub-optimal HIV viral control on activated T-cells: An Earnest Sub study
Source: AIDS. Author manuscript; Available in PMC 2024 Dec 9. (PMC7617099; doi:10.1097/QAD.0000000000003488)
Supplement: Supplementary Table 1 [file EMS200168-supplement-Supplementary_Table_1.docx]

**Supplementary Table 1. Dynamic changes in T cell populations by randomised ART group.**

|  | Randomised ART | | | | | | | | | | | | | | |
| --- | --- | --- | --- | --- | --- | --- | --- | --- | --- | --- | --- | --- | --- | --- | --- |
|  | PI/NRTI (N=69) | | | | | PI/RAL (N=71) | | | | | PImono (N=68) | | | | |
| Weeks on Second Line Therapy | 0 | 12 | 48 | 96 | 144 | 0 | 12 | 48 | 96 | 144 | 0 | 12 | 48 | 96 | 144 |
| Numbers with results | 69 | 45 | 57 | 33 | 53 | 71 | 49 | 65 | 38 | 57 | 68 | 46 | 60 | 35 | 37 |
| CD4 Cells | 103  (81 to 124), n=66 | 201  (174 to 227) | 273 (241 to 304) | 311 (269 to 352) | 412 (372 to 453) | 120  (93 to 147) , n=66 | 213 (178 to 249) | 306 (271 to 342) | 321 (260 to 383) | 423 (363 to 483) | 112  (88 to 135)  n=61 | 225 (190 to 260) | 276  (242 to 311) | 351  (255 to 446) | 378  (325 to 430) |
| CD4 % | 9  (7 to 10) , n=66 | 11  (10 to 13) | 15  (13 to 16) | 20  (18 to 22) | 21  (19 to 23) | 11  (9 to 13) , n=66 | 13  (11 to 14) | 17  (16 to 18) | 20  (17 to 23) | 20  (18 to 23) | 9  (8 to 11)  n=61 | 13  (11 to 15) | 16  (14 to 17) | 20  (17 to 23) | 21  (18 to 24) |
| CD4+ CD38+ T-cells % | 64  (59 to 68) , | 61  (56 to 66) | 56  (53 to 59) | 57  (52 to 61) | 54  (50 to 58) | 65  (61 to 69) , n=66 | 62  (58 to 67) | 56  (52 to 59) | 59  (55 to 62) | 56  (52 to 60) | 63  (59 to 67) | 55  (51 to 60) | 52  (48 to 55) | 52  (48 to 57) | 55  (51 to 59) |
| CD4+CD38+HLADR+ T-cells % | 13  (10 to 16) , | 15  (12 to 18) | 9  (6 to 11) | 12  (8 to 16) | 7  (5 to 10) | 13  (11 to 15) | 16  (13 to 19) | 8  (7 to 9) | 13  (8 to 17) | 12  (8 to 16) | 14  (11 to 16) | 14  (12 to 17) | 9  (7 to 11) | 9  (6 to 12) | 9  (6 to 13) |
| CD4+CD38+ cells | 64  (49 to 79) , n=66 | 125  (105 to 146) | 152 (134 to 171) | 182 (151 to 213) | 228 (198 to 257) | 77 (60 to 93) | 130 (106 to 153) | 170 (147 to 194) | 185 (148 to 221) | 231 (194 to 267) | 67  (52 to 82)  n=61 | 126 (102 to 150) | 144 (122 to 166) | 181  (133 to 229) | 209  (175 to 242) |
| CD4+CD38+HLADR+ cells | 13  (8 to 18) , n=66 | 28  (22 to 35) | 21  (13 to 29) | 36  (21 to 52) | 31  (17 to 45) | 13  (10 to 16) , n=66 | 29  (22 to 36) | 23  (18 to 28) | 34  (19 to 49) | 39  (25 to 53) | 13  (10 to 16)  n=61 | 28  (22 to 33) | 21  (16 to 26) | 21  (16 to 25) | 37  (16 to 59) |
| CD8 Cells | 637  (529 to 746) , n=66 | 980  (859 to 1101) | 913 (808 to 1018) | 719 (603 to 836) | 859 (768 to 951) | 687 (586 to 787) , n=66 | 1035 (865 to 1206) | 928 (798 to 1057) | 706 (615 to 797) | 894 (771 to 1016) | 750 (629 to 872)  n=61 | 979 (846 to 1112) | 882 (774 to 990) | 723  (602 to 845) | 838  (607 to 1069) |
| CD8 % | 57  (54 to 60)  n=66 | 53  (49 to 56) | 48  (45 to 50) | 44  (40 to 47) | 43  (40 to 46), n=52 | 59  (56 to 62)  n=66 | 57  (53 to 60) , n=71 | 48  (46 to 51) | 45  (42 to 49) | 43  (41 to 46) | 59  (56 to 63)  n=61 | 52  (49 to 56) | 48  (45 to 51) | 42  (38 to 46) | 42  (39 to 46) |
| CD8‎+CD38+ T-cells % | 76  (71 to 80) | 70  (64 to 76) | 55  (50 to 60) | 52  (46 to 58) | 43  (39 to 48) | 72  (68 to 77) | 68  (62 to 74) , n=71 | 50  (45 to 56) | 54  (47 to 60) | 46  (41 to 52) | 70  (65 to 76) | 59  (52 to 66) | 46  (40 to 51) | 50  (44 to 57) | 46  (40 to 51) |
| CD8+CD38‎+HLADR+ T-cells % | 22  (18 to 26) | 25  (21 to 30) | 14  (12 to 16) | 20  (14 to 25) | 13  (10 to 16) | 21  (18 to 25) | 27  (22 to 31) , n=71 | 16  (13 to 19) | 19  (14 to 25) | 18  (14 to 22) | 20  (16 to 24) | 22  (18 to 27) | 14  (11 to 17) | 19  (13 to 25) | 17  (13 to 20) |
| CD8+CD38+ cells | 456  (378 to 533) , n=66 | 683 (580 to 787) | 494 (422 to 565) | 371 (309 to 433) | 358 (315 to 401) | 491 (409 to 573)  n=66 | 718 (565 to 871) | 442 (358 to 527) | 365 (313 to 416) | 387 (329 to 445) | 518 (414 to 622)  n=61 | 578 (472 to 684) | 390 (329 to 451) | 352  (289 to 415) | 393  (260 to 525) |
| CD8+CD38+HLADR+ cells | 150  (106 to 194) , n=66 | 253 (189 to 317) | 122  (96 to 148) | 147  (99 to 195) | 113  (80 to 147) | 144 (111 to 177)  n=66 | 288 (209 to 366) | 136 (103 to 169) | 127  (89 to 164) | 148 (113 to 182) | 134 (106 to 161)  n=61 | 203 (162 to 245) | 110  (86 to 135) | 129  (82 to 176) | 143  (85 to 201) |
| CD4:CD8 ratio | 0.2  (0.1 to 0.2) , n=66 | 0.2  (0.2 to 0.3) | 0.3  (0.3 to 0.4) | 0.5  (0.4 to 0.6) | 0.5  (0.5 to 0.6) | 0.2  (0.2 to 0.2)  n=66 | 0.3  (0.2 to 0.4) | 0.4  (0.3 to 0.4) | 0.5  (0.4 to 0.6) | 0.5  (0.5 to 0.6) | 0.2  (0.1 to 0.2)  n=61 | 0.3  (0.2 to 0.3) | 0.4  (0.3 to 0.4) | 0.5  (0.4 to 0.6) | 0.5  (0.4 to 0.7) |

Note: showing means and 95% confidence intervals.
