# Supplementary material for: Impact of sub-optimal HIV viral control on activated T-cells: An Earnest Sub study
Source: AIDS. Author manuscript; Available in PMC 2024 Dec 9. (PMC7617099; doi:10.1097/QAD.0000000000003488)
Supplement: Supplementary Table 2 [file EMS200168-supplement-Supplementary_Table_2.docx]

**Supplementary table 2: VL response over time on second-line therapy, together with median (IQR) VL at each timepoint and duration in this category at each timepoint**

| VL Category | *Weeks from switch to second-line therapy* | | | |
| --- | --- | --- | --- | --- |
|  | 12  (N=140) | 48  (N=183) | 96  (N=106) | 144  (N=147) |
| Consistent viral load suppression | N =134 (96%) | N =99 (54%) | N =42 (40%) | N =56 (38%) |
| Suppressed with transient blips, N (%)  Median (IQR) VL  Median (IQR) weeks in this category | N =1 (1%)  427261  1 | N =47 (26%)  <40 [<40, <40]  24 (12-24) | N =34 (32%)  <40 [<40, <40]  60 (32-72) | N =57 (39%)  <40 [<40, <40]  96 (48-120) |
| Low-level rebound, N (%)  Median (IQR) VL  Median (IQR) weeks in this category | - | N =27 (26%)  353 [117,801]  24 (24-24) | N =13 (13%)  244 [93,563]  32 (16-48) | N =13 (13%)  184 [47, 1152]  120 (64-120) |
| High-level rebound/non-response, N (%)  Median (IQR) VL  Median (IQR) weeks in this category | N =5 (4%)  704215 [52342,797349]  0 (0-8) | N =11 (6%)  23437 [11440,295468]  24 (0-36) | N =17 (16%)  68593 [9886,122329]  32 (16-48) | N =21 (14%)  1787 [48,51044]  80 (48-96) |
