# Supplementary material for: Impact of sub-optimal HIV viral control on activated T-cells: An Earnest Sub study
Source: AIDS. Author manuscript; Available in PMC 2024 Dec 9. (PMC7617099; doi:10.1097/QAD.0000000000003488)
Supplement: Supplementary Table 3 [file EMS200168-supplement-Supplementary_Table_3.docx]

**Supplementary Table 3. Dynamic changes in T cell population by viral load classification**

| Weeks on Second Line Therapy | | **12** | **48** | **96** | **144** |
| --- | --- | --- | --- | --- | --- |
| **CD4 Cells** | | - | - | - | - |
|  | Suppressed consistently | 217  (199 to 236) | 309  (284 to 334) | 394  (312 to 476) | 431  (385 to 477) |
|  | Suppressed with transient blips | - | 279  (237 to 322) | 328  (278 to 377) | 451  (402 to 499) |
|  | low-level rebound | - | 257.65 (205.3 to 310.01) | 258 (205 to 310) | 291.23 (243 to 339) |
|  | High-level rebound/non-response | 138  (25 to 251) | 171  (112 to 229) | 193  (122 to 265) | 269  (187 to 351) |
| P comparing 4 groups (ANCOVA) | | 0.023 | 0.001 | 0.006 | 0.001 |
| P Suppressed with transient blips vs Suppressed consistently | | - | 0.094 | 0.041 | 0.99 |
| P low-level rebound vs Suppressed consistently | | - | 0.057 | 0.098 | 0.12 |
| P High-level rebound/non-response vs Suppressed consistently | | 0.034 | 0.0002 | 0.001 | 0.0004 |
| **CD4 %** | | - | - | - | - |
|  | Suppressed consistently | 12  (11 to 13) | 16  (15 to 17) | 22  (20 to 24) | 22  (20 to 23) |
|  | Suppressed with transient blips | - | 16  (15 to 18) | 20  (18 to 22) | 23  (21 to 25) |
|  | low-level rebound | - | 16  (13 to 18) | 21  (17 to 24) | 21  (17 to 25) |
|  | High-level rebound/non-response | 12  (7 to 17) | 12  (9 to 16) | 13  (9 to 16) | 14  (10 to 17) |
| P comparing 4 groups (ANCOVA) | | 0.056 | 0.007 | 0.0002 | <0.0005 |
| P Suppressed with transient blips vs Suppressed consistently | | - | 0.48 | 0.77 | 0.45 |
| P low-level rebound vs Suppressed consistently | | - | 0.86 | 0.75 | 0.76 |
| P High-level rebound/non-response vs Suppressed consistently | | - | 0.001 | <0.0005 | <0.0005 |
| **CD4+ CD38+ T-cells %** | | - | - | - | - |
|  | Suppressed consistently | 60  (57 to 62) | 55  (53 to 57) | 55  (52 to 59) | 53  (50 to 57) |
|  | Suppressed with transient blips | - | 54  (49 to 58) | 56  (52 to 60) | 55  (51 to 59) |
|  | low-level rebound | - | 52  (46 to 58) | 55  (45 to 65) | 48  (66 to 10) |
|  | High-level rebound/non-response | 61  (43 to 79) | 62  (55 to 69) | 58  (51 to 65) | 59  (53 to 66) |
| P comparing 4 groups (ANCOVA) | | 0.289 | 0.21 | 0.67 | 0.25 |
| P Suppressed with transient blips vs Suppressed consistently | | - | - | - | - |
| P low-level rebound vs Suppressed consistently | | - | - | - | - |
| P High-level rebound/non-response vs Suppressed consistently | | - | - | - | - |
| **CD4+CD38+HLADR+ T-cells %** | | - | - | - | - |
|  | Suppressed consistently | 15  (13 to 17) | 8  (7 to 10) | 9  (5 to 12) | 10  (6 to 14) |
|  | Suppressed with transient blips | - | 8  (4 to 12) | 11  (7 to 16) | 7  (5 to 9) |
|  | low-level rebound | - | 9  (6 to 12) | 10  (6 to 13) | 7  (4 to 9) |
|  | High-level rebound/non-response | 16  (10 to 22) | 13  (9 to 18) | 19  (13 to 26) | 11  (24 to 7) |
| P comparing 4 groups (ANCOVA) | | 0.56 | 0.27 | 0.026 | 0.023 |
| P Suppressed with transient blips vs Suppressed consistently | | - | - | 0.3 | 0.22 |
| P low-level rebound vs Suppressed consistently | | - | - | 0.9 | 0.25 |
| P High-level rebound/non-response vs Suppressed consistently | | - | - | 0.003 | 0.048 |
| **CD4+CD38+ cells** | | - | - | - | - |
|  | Suppressed consistently | 130 (117 to 143) | 169  (154 to 185) | 219  (176 to 263) | 230  (201 to 260) |
|  | Suppressed with transient blips | - | 153  (122 to 183) | 184  (154 to 215) | 249  (216 to 282) |
|  | low-level rebound | - | 134  (100 to 168) | 160  (120 to 200) | 196  (137 to 255) |
|  | High-level rebound/non-response | 86  (0 to 171) | 100  (72 to 128) | 107  (65 to 149) | 156  (105 to 196) |
| P comparing 4 groups (ANCOVA) | | 0.098 | 0.016 | 0.009 | 0.025 |
| P Suppressed with transient blips vs Suppressed consistently | | - | 0.32 | 0.11 | 0.452 |
| P low-level rebound vs Suppressed consistently | | - | 0.089 | 0.067 | 0.32 |
| P High-level rebound/non-response vs Suppressed consistently | | - | 0.004 | 0.001 | 0.018 |
| **CD4+CD38+HLADR+ cells** | |  |  |  |  |
|  | Suppressed consistently | 29  (25 to 32) | 23  (19 to 27) | 30  (17 to 43) | 44  (24 to 64) |
|  | Suppressed with transient blips | - | 20  (10 to 31) | 34  (18 to 50) | 32  (20 to 43) |
|  | low-level rebound | - | 20  (14 to 27) | 26  (17 to 34) | 22  (10 to 34) |
|  | High-level rebound/non-response | 20  (8 to 32) | 21  (13 to 29) | 28  (20 to 37) | 33  (22 to 44) |
| P comparing 4 groups (ANCOVA) | | 0.37 | 0.83 | 0.86 | 0.56 |
| P Suppressed with transient blips vs Suppressed consistently | | - | - | - | - |
| P low-level rebound vs Suppressed consistently | | - | - | - | - |
| P High-level rebound/non-response vs Suppressed consistently | | - | - | - | - |
| **CD8 Cells** | | - | - | - | - |
|  | Suppressed consistently | 1019  (936 to 1103) | 998  (902 to 1094) | 750  (649 to 850) | 889  (767 to 1011) |
|  | Suppressed with transient blips | - | 789  (679 to 899) | 730  (606 to 854) | 832  (735 to 929) |
|  | low-level rebound | - | 866  (713 to 1018) | 601  (483 to 718) | 697  (548 to 845) |
|  | High-level rebound/non-response | 509  (256 to 762) | 697  (387 to 1006) | 692  (538 to 847) | 1008  (619 to 1398) |
| P comparing 4 groups (ANCOVA) | | 0.092 | 0.019 | 0.241 | 0.377 |
| P Suppressed with transient blips vs Suppressed consistently | | - | 0.016 | - | - |
| P low-level rebound vs Suppressed consistently | | - | 0.39 | - | - |
| P High-level rebound/non-response vs Suppressed consistently | | - | 0.015 | - | - |
| **CD8 %** | | - | - | - | - |
|  | Suppressed consistently | 54  (52 to 56) | 49  (47 to 51) | 43  (40 to 46) | 43  (40 to 46) |
|  | Suppressed with transient blips | - | 45  (42 to 48) | 44  (40 to 48) | 40  (38 to 43) |
|  | low-level rebound | - | 50  (46 to 55) | 41  (36 to 45) | 42  (37 to 47) |
|  | High-level rebound/non-response | 48  (34 to 61) | 48  (41 to 55) | 47  (42 to 53) | 50  (44 to 55) |
| P comparing 4 groups (ANCOVA) | | 0.027 | 0.14 | 0.54 | <0.0005 |
| P Suppressed with transient blips vs Suppressed consistently | | 0.01 | - | - | 0.19 |
| P low-level rebound vs Suppressed consistently | | - | - | - | 0.87 |
| P High-level rebound/non-response vs Suppressed consistently | | 0.46 |  |  | 0.008 |
| **CD8‎+CD38+ T-cells %** | | - | - | - | - |
|  | Suppressed consistently | 65  (61 to 69) | 50  (46 to 54) | 48  (43 to 53) | 40  (36 to 44) |
|  | Suppressed with transient blips | - | 47  (40 to 53) | 50  (44 to 56) | 44  (40 to 48) |
|  | low-level rebound | - | 49  (42 to 56) | 59  (48 to 71) | 49  (38 to 60) |
|  | High-level rebound/non-response | 80  (61 to 99) | 70  (56 to 83) | 60  (50 to 71) | 59  (50 to 69) |
| P comparing 4 groups (ANCOVA) | | 0.006 | 0.011 | 0.024 | <0.0005 |
| P Suppressed with transient blips vs Suppressed consistently | | 0.003 | 0.45 | 0.27 | 0.074 |
| P low-level rebound vs Suppressed consistently | | - | 0.93 | 0.026 | 0.14 |
| P High-level rebound/non-response vs Suppressed consistently | | 0.23 | 0.002 | 0.009 | p<0.0005 |
| **CD8+CD38‎+HLADR+ T-cells %** | | - | - | - | - |
|  | Suppressed consistently | 24  (22 to 27) | 14  (12 to 16) | 13  (11 to 16) | 13  (10 to 16) |
|  | Suppressed with transient blips | - | 12  (9 to 16) | 18  (13 to 24) | 13  (10 to 15) |
|  | low-level rebound | - | 14  (10 to 17) | 24  (12 to 35) | 17  (8 to 25) |
|  | High-level rebound/non-response | 35  (32 to 38) | 27  (18 to 35) | 34  (23 to 44) | 31  (22 to 39) |
| P comparing 4 groups (ANCOVA) | | 0.029 | <0.0005 | <0.0005 | <0.0005 |
| P Suppressed with transient blips vs Suppressed consistently | | 0.03 | 0.21 | 0.12 | 0.96 |
| P low-level rebound vs Suppressed consistently | | - | 0.84 | <0.0005 | 0.23 |
| P High-level rebound/non-response vs Suppressed consistently | | 0.11 | <0.0005 | 0.029 | <0.0005 |
| **CD8+CD38+ cells** | | - | - | - | - |
|  | Suppressed consistently | 670  (597 to 744) | 489  (427 to 552) | 352  (297 to 408) | 338  (289 to 386) |
|  | Suppressed with transient blips | - | 341  (281 to 400) | 356  (302 to 410) | 348  (306 to 391) |
|  | low-level rebound | - | 436  (313 to 559) | 353  (271 to 436) | 325  (247 to 404) |
|  | High-level rebound/non-response | 405  (168 to 641) | 437  (286 to 588) | 406  (300 to 513) | 599  (371 to 827) |
| P comparing 4 groups (ANCOVA) | | 0.39 | 0.1 | 0.91 | <0.0005 |
| P Suppressed with transient blips vs Suppressed consistently | | - | - | - | 0.76 |
| P low-level rebound vs Suppressed consistently | | - | - | - | 0.55 |
| P High-level rebound/non-response vs Suppressed consistently | | - | - | - | <0.0005 |
| **CD8+CD38+HLADR+ cells** | | - | - | - | - |
|  | Suppressed consistently | 251  (213 to 289) | 133  (112 to 155) | 96  (70 to 122) | 106  (86 to 127) |
|  | Suppressed with transient blips | - | 86  (62 to 110) | 133  (87 to 180) | 108  (80 to 136) |
|  | low-level rebound | - | 121  (78 to 164) | 134  (72 to 195) | 104  (48 to 159) |
|  | High-level rebound/non-response | 179  (92 to 266) | 192  (53 to 330) | 229  (137 to 320) | 299  (191 to 407) |
| P comparing 4 groups (ANCOVA) | | 0.54 | 0.07 | 0.032 | <0.0005 |
| P Suppressed with transient blips vs Suppressed consistently | | - | - | 0.18 | 0.95 |
| P low-level rebound vs Suppressed consistently | | - | - | 0.31 | 0.54 |
| P High-level rebound/non-response vs Suppressed consistently | | - | - | 0.003 | <0.0005 |
| **CD4:CD8 ratio** | | - | - | - | - |
|  | Suppressed consistently | 0.3  (0.2 to 0.3) | 0.4  (0.3 to 0.4) | 0.6  (0.5 to 0.7) | 0.5  (0.5 to 0.6) |
|  | Suppressed with transient blips | - | 0.4  (0.3 to 0.5) | 0.5  (0.4 to 0.6) | 0.6  (0.5 to 0.7) |
|  | low-level rebound | - | 0.3  (0.3 to 0.4) | 0.5  (0.4 to 0.6) | 0.5  (0.4 to 0.6) |
|  | High-level rebound/non-response | 0.3  (0.1 to 0.4) | 0.3  (0.2 to 0.4) | 0.3  (0.2 to 0.4) | 0.3  (0.2 to 0.4) |
| P comparing 4 groups (ANCOVA) | | 0.71 | 0.37 | 0.02 | <0.0005 |
| P Suppressed with transient blips vs Suppressed consistently | | - | - | 0.31 | 0.15 |
| P low-level rebound vs Suppressed consistently | | - | - | 0.98 | 0.3 |
| P High-level rebound/non-response vs Suppressed consistently | | - | - | 0.028 | 0.002 |

Note: showing means and 95% confidence intervals. ANCOVA corrected for baseline values.
